# Ebola in the Eastern Democratic Republic of Congo: One Health approach to infectious disease control

**DOI:** 10.1016/j.onehlt.2019.100117

**Published:** 2019-12-05

**Authors:** Franck katembo Sikakulya, Olivier Mulisya, Dalton Kambale Munyambalu, Gabriel Kambale Bunduki

**Affiliations:** a Faculty of Medicine, Université Catholique du Graben, Butembo, Congo; b Department of Surgery, Kampala International University, Western-Campus, Ishaka, Uganda; c Department of Obstetrics and Gynecology, FEPSI Hospital, Butembo, Congo; d Department of Internal Medicine, Kampala International University, Western-Campus, Ishaka, Uganda; e Department of Infectious diseases, Université Catholique du Graben, Butembo, Congo

**Keywords:** Ebola, One health, Infectious disease control, Democratic Republic of Congo, DRC, Democratic Republic of Congo, EVD, Ebola virus disease, MoH, Ministry of Health, WHO, World Health Organization, SARS, Severe Acute Respiratory Syndrome, MERS, Middle-East Respiratory Syndrome, IPC, Infections Prevention and Control, EHPs, Environmental Health Practitioners

## Abstract

The Democratic Republic of Congo (DRC) is facing its tenth outbreak of Ebola virus disease (EVD), in North-Kivu and Ituri provinces. This is the second most deadly EVD outbreak in history, after the one that occurred in West Africa in 2014. The DRC Ministry of Health (MoH), supported by the World Health Organization (WHO) and a range of regional and international partners, are implementing EVD response plans in these affected areas such as screening of suspect cases at points of entry, case detection, contact tracing, laboratory testing, case management and infection prevention and control, safe and dignified burials, ring vaccination (this involves vaccination of infected individuals, direct contacts of infected individuals and contacts of their contacts), and therapeutics, community mobilization and free access to healthcare services. Despite these efforts, there has been a sharp rise in the number of confirmed cases within the identified affected areas, and due to a number of challenges unique to DRC, there has been an expansion in the geographical extent of transmission. The significance of the proximity of these regions to wildlife and the Virunga National Park is questionable in the EVD transmission dynamics. The close interaction between human, animal, and environmental factors, in combination with high population movement due to regular rebel attacks in these regions, suggest the need for the integration of the One Health approach in the holistic response plans for control and prevention of EVD. This paper seeks to highlight the implications and importance of a One Health–based approach into the infectious diseases control program implementation in DRC.

## 1 Introduction

The Democratic Republic of Congo (DRC) has been facing its tenth outbreak of Ebola virus disease (EVD) since 1 August 2018. This outbreak is currently ongoing in the North-Kivu and Ituri provinces of DRC. Previous outbreaks in DRC occurred in remote and hard-to-reach areas, while the ongoing outbreak is occurring in urban environments. Furthermore, there are other unique challenges during this outbreak, such as community resistance and security issues due to the epicentre being in a conflict zone. In the history of EVD outbreaks, this has been the second most deadly after the one that occurred in West Africa in 2014. The DRC is located in central Africa, neighboring with nine countries (Angola, Burundi, Central African Republic, Republic of Congo, Rwanda, South Sudan, Tanzania, Uganda, and Zambia). A majority of the population from North-Kivu and Ituri provinces travel for trade and business between their neighboring countries in the East (Burundi, Rwanda, Tanzania, and Uganda). Hence, controlling the outbreak in those two proviences would prevent its spread to these countries.

The Ebola virus genus belongs to the *Filoviridae* family and has five species: *Zaire ebolavirus, Sudan ebolavirus, Reston ebolavirus, Taï Forest ebolavirus, and Bundibugyo ebolavirus* [[1], [2], [3], [4]].

This tenth EVD outbreak is caused by the *Zaire ebolavirus* [5]. The natural reservoir for Ebola has yet to be confirmed, however, bats are considered to be the most likely candidate species [6]. Three types of fruit bats (*Hypsignathus monstrosus*, *Epomops franqueti,* and *Myonycteris torquata*) were found to possibly carry the virus without getting sick [7]. Between people, Ebola disease spreads only by direct contact with the blood or other body fluids of a person who has developed symptoms of the disease or by contact with contaminated surfaces and materials [8].

As of 17 November 2019, a total of 3296 EVD cases were reported, including 3178 confirmed and 118 probable cases, of which 2196 individuals died and 1075 survived (overall case fatality ratio 67%) in the eastern DRC [9]. Most of these cases have been reported in the community, however a few cases have also been reported in healthcare settings [9]. The DRC Ministry of Health (MoH), supported by the World Health Organization (WHO) and a range of regional and international partners are implementing EVD response plans in these affected areas. A package of interventions, such as screening of suspect cases at points of entry, case detection, contact tracing, laboratory testing, case management and infection prevention and control, safe and dignified burials, ring vaccination (This involved vaccination of infected individuals; direct contacts of infected individuals, and contacts of those contacts); and therapeutics, laboratory facilities with capacity for EVD confirmatory testing, safe and dignified burials, community mobilization, and free access to healthcare services, have been used for containing and eliminating this deadly disease [10,11]. Conducting contacts tracing and early symptoms detection in this unstable setting remains a challenge. The population is highly mobile with individuals often hiding, refusing to subject themselves to follow-up examinations, or traveling to distant homes in heavily forested areas and other destinations [5]. The rVSV-ZEBOV experimental vaccine has been offered to healthcare and frontline workers, case contacts, and contacts of contacts, and the GeneXpert diagnostic test tool has been introduced for rapid diagnosis of the Zaire strain of EVD [9,12,13]. From 8 August 2018 to 22 July 2019, 171,052 people have been vaccinated using the rVSV-ZEBOV-GP vaccine with an efficacy of 97.5% according to the WHO [5,14]. Despite these efforts, the number of cases has increased sharply.

Logistical challenges due to poor infrastructure continue to affect surveillance, case detection and confirmation, contact tracing, access to vaccines and therapeutics, risk communication, and community engagement activities. In the complexities of EVD transmission, the proximity of these regions to wildlife species and the Virunga National Park is unclear. Due to regular rebel attacks in these areas, the close interaction between human, animal, and environmental factors, in combination with high population movement indicates the need for further incorporation of the One Health approach into the integrated EVD response plans [5].

Difficulties in contact tracing are encountered due to community resistance in this area [11]. There have been many instances of attacks against EVD response teams and facilities, exacerbating the spread of the virus [5]. The EVD outbreak was declared in the neighboring country Uganda, with two fatal cases confirmed to date. These cases are linked to the ongoing outbreak in eastern DRC [15].

A One Health approach would bring together all stakeholders to address the interconnected factors responsible for the spread of Ebola. This would include cultural practices, vector control, and animal and environmental conditions, and could help to control this Ebola outbreak and to prevent the emergence of infectious diseases in the future.

This paper seeks to highlight the implications and importance of a One Health–based approach to the infectious diseases control program implementation in DRC. We will discuss how Ebola moves from animal to human, the potential infection transmission events which led to the current EVD outbreak in DRC, and the Ebola virus environmental health hazard.

## 2 One health approach in disease control

Outbreaks of zoonotic diseases such as EVD and Severe Acute Respiratory Syndrome (SARS) are a reminder of the inextricable links between humans, animals, and the environment. These linkages call for new tools for cooperation and collaboration between public health professionals and conservationists to better address the vital issues of these emerging infectious diseases [16].

It is estimated that, globally, approximately one billion cases of illness and millions of deaths occur every year from zoonoses. Up to 60% of emerging infectious diseases that are reported globally are zoonoses. Understanding the complex inter-relationship between humans, animals, and the environment is important for not just designing and implementing specific and effective interventions for EVD outbreak response, but also for the control and prevention of other zoonotic diseases such as rabies, Middle-East Respiratory Syndrome (MERS)-corona, influenza, and other Ebola viruses, in situations where the environment is shared [17]. Factors that influence this inter-relationship include ecology, demographics, behavioral and socioeconomic changes, and civil conflicts in this particular region of eastern DRC.

As a zoonosis, Ebola occasionally spills over to humans, apes, and possibly other animals. Fruit bats belonging to the *Pteropodidae* family are thought to be hosts of Ebola virus, but in natural conditions, there has been no isolation of the virus from bats. Directly, humans are likely infected either by touching or consuming sick or dead infected forest animals, including infected fruit bats and nonhuman primates [18].

Direct contacts with blood, secretions, or other body fluids from infected humans can explain secondary human-to-human transmission, especially when health workers do not respect measures of infections prevention and control (IPC), which explains why nosocomial transmission occurs frequently before the outbreak is identified. Caring for the sick or handling dead bodies (e.g. during traditional funerals) is also associated with a particularly high risk of disease transmission between humans, which can occur frequently before the outbreak is identified. Sexual transmission by survivors of EVD has also been reported [19,20].

The re-emergence of zoonoses have their roots in many causes [21]. The global economy has enabled the rapid spread of people, animals, plants, and agricultural products across the world. This mobility has contributed to more frequent outbreaks of zoonotic diseases and infections of uninformed populations. Improved coordination among key ministries (health, livestock, fisheries, wildlife, environment, disaster response, and partners) guided by the One Health approach is necessary to match responses to disease ecology [22,23]. This is done by conducting in-depth joint outbreak investigations and response activities by establishing a One Health coordination unit. This unit should bridge the animal and human health sectors by deploying an epidemiologist from each ministry in order to maintain collaboration at the animal and human health interface with the goal of better prevention and control of zoonosis. Furthermore, an ecologist should be added to the unit to ensure that environmental risks are adequately addressed in emerging disease control. Such collaboration has been applied successfully in other countries such as Kenya [24] and India [25] by applying a One Health-based approach for controlling diseases.

One Health is an interdisciplinary, collaborative effort to attain optimal health for people, animals, and the environment. WHO describes One Health as “an approach to designing and implementing programs, policies, legislation, and research in which multiple sectors communicate and work together to achieve better public health outcomes” [26].

During the EVD outbreak that occurred in Sierra Leone, Liberia, and Guinea, several factors contributed to gaps in the strategies implemented to control the outbreak in these countries [27].

There were insufficient monitoring and ecological modeling of zoonotic infection and transmission, insufficient systems for rapid dissemination of community education about the ecological aspects of disease outbreak and management, and insufficient resources committed to enhancing food security that would limit environmental encroachment and exposure to zoonotic diseases in the wild. The association of ecological and wildlife management during the outbreak in these countries played a major role in controlling the incidence of new cases by using the theory that better management might begin with community education about the involvement of environment and animal factors in the genesis of Ebola [27,28]. Thus, a One Health (ecological, demographic, behavioral, and socioeconomic) approach could help to control an outbreak.

The West African EVD outbreak presented another opportunity to incorporate the One Health paradigm into infectious disease control. Specifically, data gathered from sustained surveillance of local wildlife led to updated data on biodiversity of the bats and primates found in the region. Both of these animal groups could be related to Ebola virus infection events in both animals and humans. Human-animal interface zones with a high propensity for contact and consequent exposure to infectious diseases including EVD were identified. Community engagement and socioeconomic re-orientation, with a critical analysis of contact frequency and risks between reservoir hosts and susceptible animals or humans needed to be considered [21].

In Uganda, during previous outbreaks, including zoonoses such Ebola and Marburg fever, the environmental health practitioners (EHPs) working in the One Health team contributed immensely to reducing morbidity and mortality from these outbreaks [29]. Their duties included inspection of animals before slaughter (ante mortem) and meat in abattoirs (post mortem), inspection of meat in butcheries, destruction of condemned meat, disease surveillance, outbreak investigation and control of zoonosis, control of vectors and vermin such as rats, fleas, mosquitoes, and monkeys, health education on pertinent issues such as vaccination of dogs, and food safety including meat and milk. They also played an important role in prevention, detection, and abatement of microbial and chemical pollution of land, air, and water sources that have created new threats to the health of both animals and humans. EHPs carry out house to house inspections on water, sanitation, and hygiene, hence are involved in abating risk factors at households that could pose a threat to public health. Such threats could be linked to the environment, as well as to animals [30].

Considering the sociopolitical aspects of the region and ecological aspects in the Ebola response team, the DRC government's implementation of strategies may have an impact in escalating the ongoing outbreak response. Integrating conservationists in the prevention of Ebola should play a major role in interrupting predictable chains of transmission.

Maintenance and extension of the classic control modalities, such as epidemiological surveillance, clinical medicine, and clinical virology are essential. In addition, new tools such as mathematical modeling, remote sensing, and ecologically based approaches are also important [30]. Mathematical models can project how infectious diseases progress to show the likely outcome of an epidemic and help inform public health interventions. Models use some basic assumptions and mathematics to find parameters for various infectious diseases and use those parameters to calculate the effects of possible interventions, such as mass vaccination programs. Research that applies an environmental approach to emerging viral diseases is particularly complex. Remote sensing data enable scientists to study the earth's biotic and abiotic components. The causes underlying the rise of disease are at the macro (socio-cultural) and micro (cellular and molecular) levels, but they are indeed acting over individuals, populations, and communities. There are several lines of evidence on the relationship between natural ecosystems' intervention and re/emergence of diseases produced by bacteria, parasites and viruses. Specifically, in order to understand viral emergence it is important to understand the sylvatic cycle of viruses, the transition to human populations, the relationship between vectors, pathogens and reservoirs in wildlife ecosystems, the change in the distribution of vectors and reservoirs after natural habitat fragmentation, and how these conditions are generating potential new roles and ecological niches for species. Wild ecosystems historically disturbed by agricultural and industrial activities with changes in biotic and abiotic factors (water bodies distribution, soil profiles, plant coverage, breeding microclimate, vertebrate and invertebrate populations, etc.), constitute new selective pressures for pathogens and therefore new opportunities for adaptation. It allows vectors/reservoirs to exploit the new resources, favouring viral contact with potentially new host populations (e.g. humans) [31]. From all these aspects, the integrated approach of One Health shows its usefulness in controlling re-emerging infectious diseases.

## 3 Limitations of implementing one health approach in the Democratic Republic of Congo

Successfully implementing the One Health approach also requires a global network of qualified individuals working locally, regionally, nationally and internationally to share information, conduct disease surveillance in human and animal populations, monitor the environment, improve food safety and security, and communicate effectively to the public. Such implications need participation of the local population and stability of the area or the nation to make the One Health approach effective. The eastern part of DRC where the EVD outbreak is ongoing is facing some challenges such as community resistance and security issues due to the epicentre being in a conflict zone. This area has experienced successive wars and rebel attacks over the past 20 years, which has led to increased movement of the population. Such conditions in this area may hamper the implementation and delivery of a One Health approach in prevention and control of infectious disease. Effective implementation of One Health approach in both its eastern parts, as well as the rest of DRC, requires the DRC government's support in establishing security and stability in the region.

## 4 Conclusion

As the number of new Ebola cases increases in the eastern DRC, issues involving human, animal, and environmental health have gained global attention. National and regional public health sectors should give priority to deploying surveillance systems and enhanced diagnostic tools regarding emerging pathogens. A broad collaboration among clinicians, public health workers, veterinarians, and veterinary public health officials is necessary for prompt response strategies and ensuring the prevention and management of zoonosis. The reduction of zoonotic risks in farms should be a priority in order to improve the overall health of humans and animals. The ongoing EVD outbreak in eastern DRC gives an insight into the integration of the One Health approach into the implementation of infectious diseases control programs in DRC.

## Ethics approval and consent to participate

Not applicable.

## Consent for publication

Not applicable.

## Availability of data and material

Not applicable.

## Funding

This work received no specific grant from any funding agency in the public, commercial, or not-for-profit sectors.

## Authors' contributions

FKS participated in the conception and design of the study, literature search and drafted the manuscript.

OM and DKM participated in literature search.

GKB participated in the conception and design of the study, literature search, helped in drafting the manuscript and critically reviewed it for its scientific contents.

All authors read and approved the submitted manuscript.

## Declaration of Competing Interest

Authors declare no conflicts of interest exist.
